# Single-cell transcriptomics reveals apolipoprotein A4-mediated metabolic-immune reprogramming in lymphocytes during early obesity-related chronic kidney disease: Lymphocyte immunity and metabolism in early obese CKD mice

**DOI:** 10.3724/abbs.2025171

**Published:** 2025-09-25

**Authors:** Yang Wei, Ting Zhang, Yingying Jin, Xiaohuan Liu, Jinting Zhou, Na Huang, Yiying Wang

**Affiliations:** 1 Department of Pathology the Second Affiliated Hospital of Xi’an Jiaotong University Xi’an 710004 China; 2 Core Research Laboratory the Second Affiliated Hospital of Xi’an Jiaotong University Xi’an 710004 China; 3 Department of Radiation Therapy the Second Affiliated Hospital of Xi’an Jiaotong University Xi’an 710004 China; 4 National & Local Joint Engineering Research Center of Biodiagnosis and Biotherapy Precision Medical Institute the Second Affiliated Hospital of Xi’an Jiaotong University Xi’an 710004 China; 5 Key Laboratory of the Ministry of Public Health for Forensic Sciences Western China Science & Technology Innovation Harbor Xi’an 710100 China; 6 The School of Public Health Hebei Medical University Shijiazhuang 050011 China

**Keywords:** chronic kidney disease, obesity, immune, metabolism, single-cell RNA sequencing, Apoa4

## Abstract

Obesity-induced metabolic inflammation is a key driver of chronic kidney disease (CKD), with immune dysregulation, particularly among lymphocytes, contributing to early disease pathology. To explore the role of apolipoprotein A4 (Apoa4) in regulating immune cell metabolism and function, we establish high-fat diet-induced obese (DIO) models using wild-type and *Apoa4*-knockout (KO) mice. KO mice exhibit exacerbated insulin resistance and renal lipid accumulation. Single-cell RNA sequencing reveals that *Apoa4* deletion remodeled the renal immune-metabolic landscape. This remodeling broadly compromises the immune functions of T, NK, and B cells, even as it expands the proportions of cytotoxic Gzma ^+^ NK cells and Derl3 ^+^ plasma cells. Mechanistically, *Apoa4* deletion aggravates metabolic dysregulation and oxidative stress and downregulates the expression levels of key effector genes, including *Ifng* and *Il1b*. Furthermore, the regulatory network activities of key transcription factors, such as *Lef1* and *Runx3* in Cd8 ^+^ T cells; *Irf8*, *T-bet*, and *Eomes* in NK cells; and *Tcf4*, *Lmo2*, and *Xbp1* in B cells, are perturbed. CellChat analysis predicts disruptions in pro-inflammatory (IFN-II and IL-1), immunoregulatory (FASLG), and metabolic regulatory (ENHO and ANGPTL) signaling, alongside enhanced IL-2-mediated suppression. These findings are corroborated by flow cytometry, immunofluorescence staining, and qPCR. Our results establish Apoa4 as a crucial regulator of lymphocyte metabolic and immune homeostasis in the early stages of obesity-associated CKD.

## Introduction

Obesity-induced metabolic inflammation, insulin resistance and lipid deposition in the kidney are major contributors to the onset and progression of obesity-related chronic kidney disease (CKD) [ 1, 2]. The increasing prevalence of obesity-related CKD has become a major global public health challenge [3]. The immune system plays a crucial role in maintaining immunological homeostasis in healthy kidneys, and its dysregulation, whether overactive or underactive, accelerates disease progression [4]. Recent advances in single-cell RNA sequencing (scRNA-seq) have revealed the intricate diversity of immune cell populations in both healthy and diseased kidneys [ 5– 7], providing unprecedented insights into the heterogeneity of immune cells and their diverse roles in inflammation and tissue remodeling. Immune cell activation and function are tightly regulated by environmental cues and intercellular interactions. For example, tissue-resident memory T cells and B cells rapidly activate and exhibit effector functions upon re-encountering antigens [ 8, 9], whereas natural killer (NK) cells respond to stimulatory signals, becoming activated and directly exerting cytotoxic effects on target cells. These cells release pro-inflammatory mediators such as interferon-γ (IFN-γ) and tumor necrosis factor (TNF), which modulate innate immune cells, underscoring the critical role of lymphocytes in early inflammatory kidney disease [10]. The macrophage phenotype is dynamically regulated by the microenvironment, with M1 and M2 macrophages playing opposing roles in renal inflammation [11]. In CKD, immune cell activation is closely linked to the activation of signaling pathways, such as the MAPK signaling, NLRP3 inflammasome signaling, PI3K/Akt signaling, NF-κB signaling, JAK-STAT signaling, and Toll-like receptor signaling pathways [12].

Metabolic pathways are fundamental to immune cell function and influence immune cell proliferation, differentiation, and activation. Under healthy conditions, glucose, fatty acid, and amino acid metabolism maintain immune metabolism homeostasis. However, in diseases, including obese kidneys, disruption of cellular metabolism leads to aberrant immune responses [ 13– 15]. Aerobic glycolysis appears to drive the activation of most lymphoid and myeloid cells, whereas oxidative phosphorylation predominates in regulatory, resting, and memory immune cells [16]. Importantly, lipids play dual roles as both metabolic fuels and signaling molecules in immune regulation. The dynamic processes of extracellular lipid uptake, membrane lipid remodeling and intracellular lipid synthesis/catabolism collectively orchestrate immune cell function and disease development. Although fatty acid oxidation (FAO) has been identified as a crucial energy source for both tissue-resident memory CD8 ^+^ T cells [17] and germinal center B cells, the detailed mechanisms by which FAO regulates B-cell function remain to be fully elucidated [18]. Apolipoprotein A4 (Apoa4) is a lipid-binding protein induced by long-chain fatty acid (LCFA) absorption in the small intestine that circulates via chylomicrons and exerts pleiotropic effects through the following receptor systems: (1) the nuclear receptor NR4A1/NR1D1-mediated SERPINA3 induction, which is linked to anti-inflammatory responses and CKD [19]; (2) αIIbβ3 integrin-dependent inhibition of platelet aggregation and thrombosis; and (3) LRP1-regulated metabolic homeostasis [20] and numerous other unidentified receptors. By interacting with these receptors, Apoa4 plays a significant role in lipid metabolism, reverse cholesterol transport, atheroprotection, platelet aggregation and thrombosis, glucose homeostasis [21] and metabolic inflammation [22], and may also be involved in the inflammatory regulatory mechanisms of kidney injury [23]. Clinical studies have revealed that elevated Apoa4 concentrations correlate with attenuated inflammation in moderate CKD patients, suggesting that Apoa4 not only serves as an independent risk marker of reduced inflammation in CKD patients but also may actively participate in the pathophysiological process of CKD through its anti-inflammatory mechanisms [24]. These findings highlight the therapeutic potential of Apoa4 in renal diseases, providing a novel potential target for the clinical management of CKD. However, the precise mechanisms underlying the regulatory effects of Apoa4 on metabolic–immune interactions are poorly understood.

In the present study, we employed scRNA-seq to characterize renal immune cells in diet-induced obese (DIO) mice and compared their functional and metabolic profiles between wild type (WT) and *Apoa4* knockout mice. Our data demonstrate that Apoa4 deficiency exacerbates metabolic and immune disorders, highlighting its potential role in maintaining renal metabolic-immune homeostasis during early obesity-related CKD.

## Materials and Methods

### Diet-induced obese (DIO) mouse models

*Apoa4*-KO (KO) mice on a C57BL/6J background, generously provided by Professor Patrick Tso (Metabolic Diseases Institute, University of Cincinnati, Cincinnati, USA), were used in this study. All the mice were bred in a specific pathogen-free facility with a 12/12-h light/dark cycle. To induce obesity, six-week-old male C57BL/6J WT and KO mice were fed with a high-fat diet (HFD; 60% kcal from fat) for 16 weeks. At the end of the feeding period, the mice were anaesthetized with isoflurane (Sigma-Aldrich, Shanghai, China), followed by transcardial perfusion with phosphate-buffered saline (PBS) until complete exsanguination. Kidney tissues were then harvested for subsequent analysis. The animal study protocol was approved by the Biomedical Ethics Committee of the Medical School of Xi’an Jiaotong University in accordance with ethical principles, with approval numbers XJTUAE2023-2163 and XJTUAE2023-2164.

### Preparation of single-cell suspensions from kidneys

Single-cell suspensions were prepared with modifications to a previously described method [25]. Kidneys from DIO WT and KO mice ( *n* = 3 per group) were weighed, minced into ~1 mm³ cubes, and incubated with 100 U/mL collagenase II at 37°C for 20 min with gentle agitation. The enzyme was neutralized with an equal volume of RPMI 1640 containing 10% fetal calf serum. The mixture was filtered through a 40-μm nylon sieve to remove debris and centrifuged at 50 *g* for 5 min to reduce parenchymal cells. The supernatant was treated with red blood cell lysis buffer to remove residual red blood cells. The resulting single-cell suspensions were used for scRNA-seq.

### Single-cell RNA sequencing and data analysis

Single-cell libraries were prepared using the 10× Genomics platform (Pleasanton, USA) and sequenced on an Illumina NovaSeq 6000 by BGI (Shenzhen, China). The raw sequencing data were processed using Cell Ranger v2.1.0 to generate FASTQ files. Low-quality cells were excluded, and further analysis was conducted using Seurat [26]. The WT and KO datasets were integrated, and the cells were clustered at a resolution of 0.5. *Ptprc ^+^* ( *Cd45 ^+^*) immune cells were isolated, revealing 14 immune cell types. Marker genes were identified using FindConservedMarkers, and differentially expressed genes (DEGs) between the KO and WT subsets were identified using FindMarkers. Enrichment analysis of positively expressed marker genes was performed via the Kyoto Encyclopedia of Genes and Genomes (KEGG) [ 27– 29 ] to explore subset features and functions. Gene set enrichment analysis (GSEA) of DEGs via KEGG and Gene Ontology (GO) analysis was performed to observe feature or functional changes between the KO and WT subsets. All enrichment analysis was carried out using the R package ClusterProfiler [30]. Transcription factors and their target genes as regulons (regulatory network) were reconstructed, and the activity of these regulons was assessed using SCENIC (version 1.2.0) [31]. Cell-cell communication was analyzed using CellChat v0.0.2 [32] .

### Fluorescence-activated cell sorting (FACS)

Kidney tissues were digested, filtered through 40-μm sieves, and treated with red cell lysis buffer. The cells were stained with CD3-FITC and NK1.1-PerCP antibodies (Thermo Fisher Scientific, Waltham, USA) and analyzed using a BD FACSCalibur flow cytometer (BD Biosciences, San Jose, USA). Data analysis was performed with FlowJo software (Tree Star, Ashland, USA).

### Immunofluorescence staining

Kidney tissues were fixed in 4% paraformaldehyde, paraffin-embedded, and sectioned. After deparaffinization, antigen retrieval was performed via citrate buffer (pH 6.0), followed by blocking with 5% BSA. The sections were incubated overnight at 4°C with primary antibodies at 1:200 and subsequently with secondary antibodies (Thermo Fisher Scientific) at room temperature for 2 h. The nuclei were counterstained with DAPI, and sections were mounted with antifade medium. Imaging was performed via a Pannoramic MIDI scanner (3DHISTECH, Budapest, Hungary) or Nikon C2 confocal microscope (Nikon, Tokyo, Japan). The primary antibodies used included CD3 monoclonal antibody (Thermo Fisher Scientific) and HOPX polyclonal antibody (Proteintech, Wuhan, China).

### Kidney histology

Harvested kidneys were fixed in 10% formalin, embedded in paraffin, and cut into 4-μm sections. The sections were then deparaffinized and rehydrated through a graded ethanol series to water, followed by staining with Haematoxylin and Eosin (H&E). Subsequently, the stained sections were dehydrated, cleared, and cover-slipped for mounting. Finally, whole-slide imaging was performed using a Zeiss Axio Scan Z1 slide scanner (Zeiss, Wetzlar, Germany).

### Real-time-quantitative PCR analysis

Kidney cells were isolated from WT and KO mice. Total RNA was extracted from kidney cells using TRIzol (Invitrogen, Carlsbad, USA). First-strand cDNA synthesis was carried out via the PrimeScript RT Reagent Kit with gDNA Eraser (#RR047A; Takara, Dalian, China). Real-time quantitative PCR (qPCR) was conducted on a QuantStudio 5 (Applied Biosystems, Foster City, USA) with TB Green Premix Ex Taq II (#RR820A; Takara) following standard protocols. All sequences of primers used in this study are presented in Supplementary Table S1.

### Statistical analysis

The data were analyzed via unpaired two-tailed Student’s *t* test or ANOVA, as appropriate, with PRISM 8.0. Data are presented as the mean ± SEM. *P* < 0.05 was considered statistically significant.

## Results

### *ApoA4* deletion exacerbates metabolic disorders in DIO mouse kidneys

**Figure 1:**
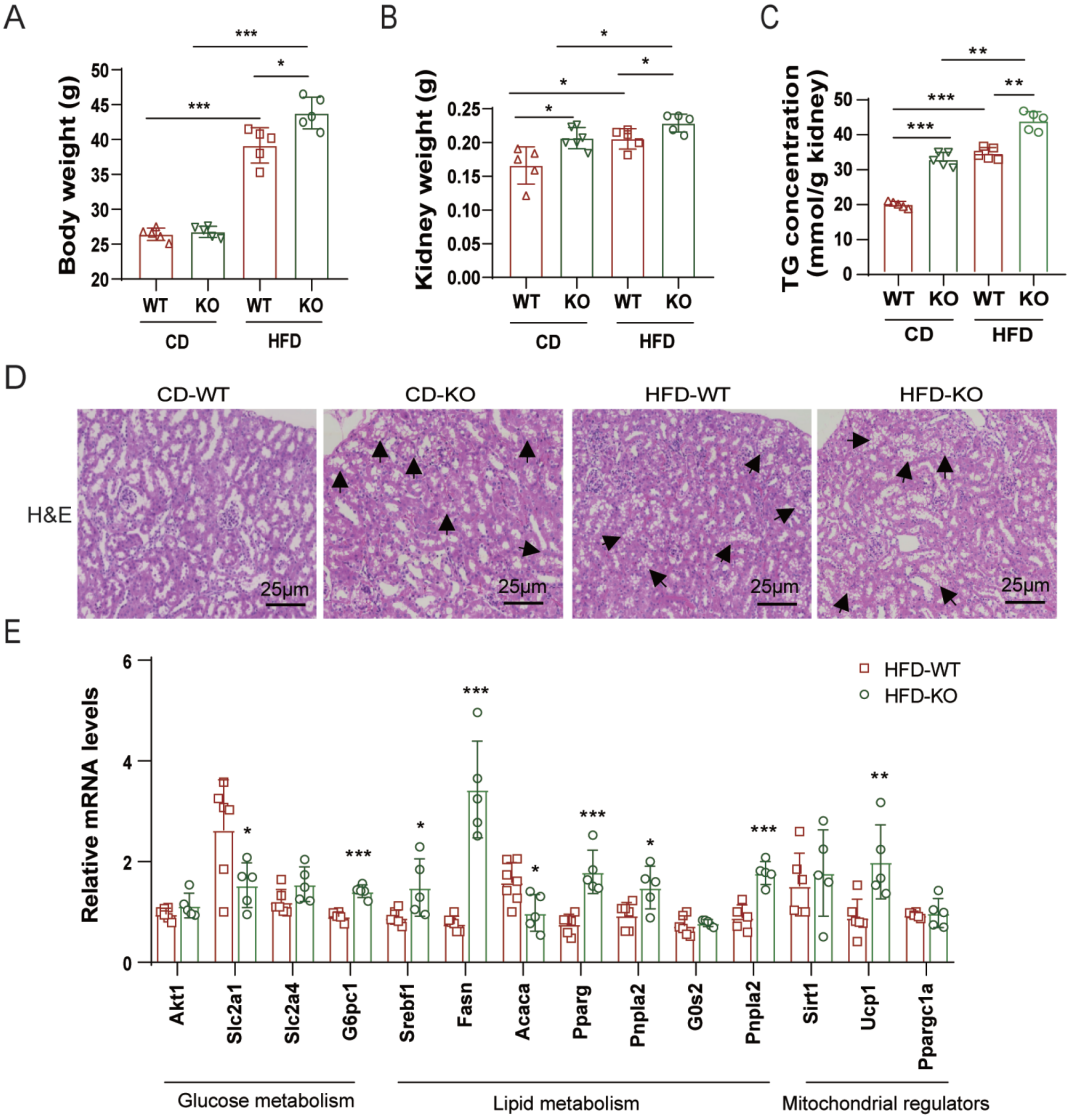
Figure 1 *ApoA4* deletion aggravates renal metabolic dysregulation in DIO mice KO mice and WT mice were fed with a high-fat diet (HFD, HFD-KO and HFD-WT groups) or a chow diet (CD, CD-KO and CD-WT groups) for 16 weeks (5 mice in each group). (A) Body weight. (B) Kidney weight. (C) Triglyceride (TG) concentration. (D) H&E staining revealed that more vacuoles were present in the proximal tubules of the KO mice than in the control mice. The short arrows indicate significant vacuoles localized in proximal tubules. (E) Relative mRNA levels of key genes involved in glucose metabolism, lipid metabolism, and mitochondrial function were quantified by RT-qPCR. *P < 0.05; **P < 0.01; *** P < 0.001.

To investigate the impact of Apoa4 on renal immune cells in obese mice, we established a high-fat DIO model using *Apoa4* KO and WT mice. Both groups developed metabolic dysfunction, including impaired glucose tolerance, reduced insulin sensitivity, and increased body/kidney weights, confirming successful obesity induction [ 33, 34]. Compared with WT mice, KO mice presented further increases in these metabolic parameters ( Figure 1A,B and Supplementary Figure S1). Notably, renal triglyceride (TG) levels were significantly higher in KO mice than in WT mice, and H&E staining revealed greater lipid vacuole deposition in the proximal tubules ( Figure 1C,D), indicating more severe renal lipid accumulation, a key feature of obesity-induced kidney damage. RT-qPCR analysis revealed substantial alterations in the expressions of key regulatory genes governing glucose metabolism, lipid homeostasis, and mitochondrial function ( Figure 1E), collectively demonstrating that Apoa4 deficiency exacerbates obesity-induced renal metabolic dysregulation.

### scRNA-seq identifies renal immune cell types in DIO mice

**Figure 2:**
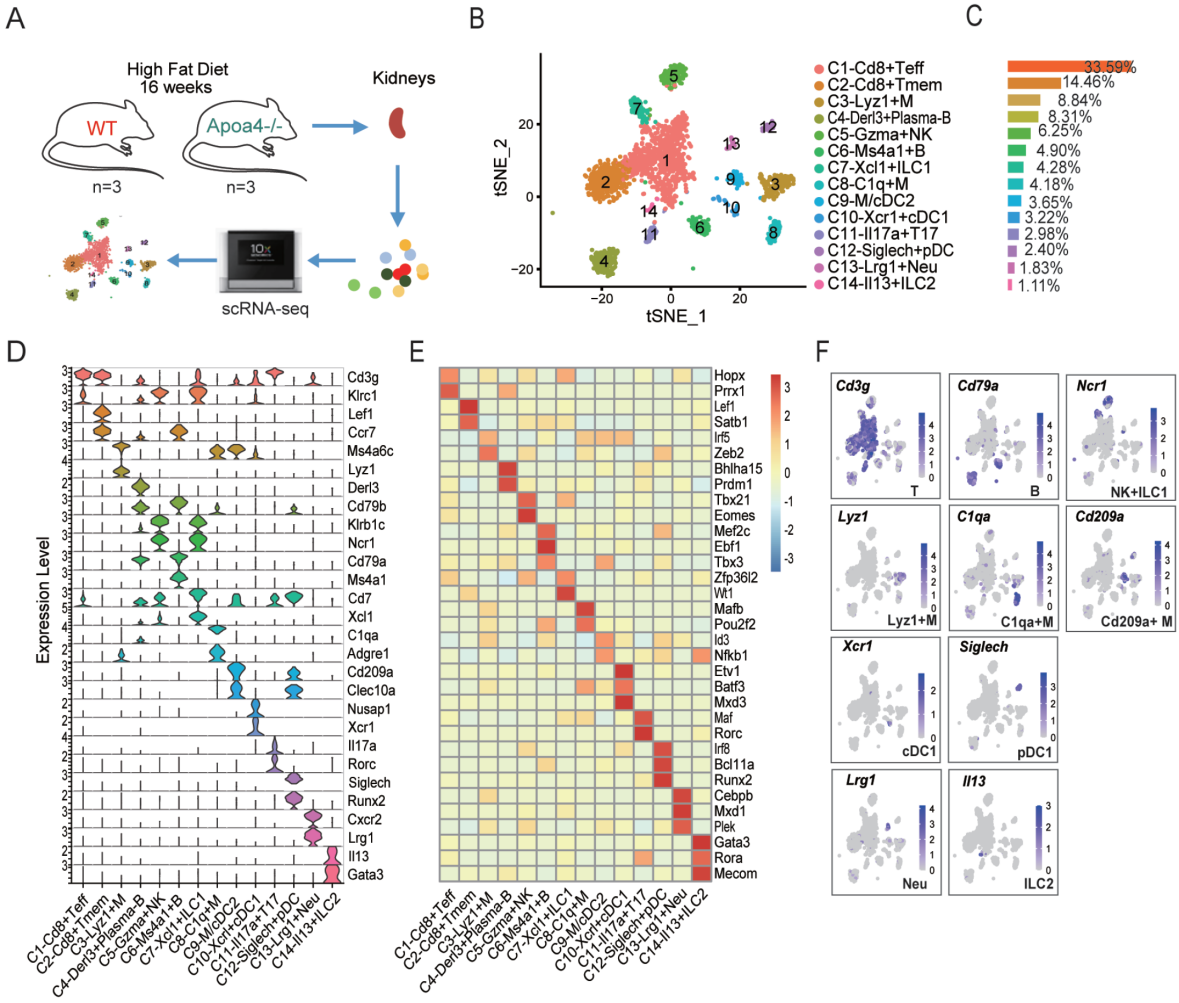
Figure 2 Immune cell landscape in DIO-mouse kidneys revealed by scRNA-seq (A) Schematic overview of the study design. (B) Clustering of immune cells from KO and WT kidneys. Teff: effector T lymphocytes; Tmem: memory T cells; B: B lymphocytes; Plasma-B: plasma cells; ILC: innate lymphoid cells; NK: natural killer cells; M: macrophages; cDC: conventional dendritic cells; pDC: plasmacytoid dendritic cells; Neu: neutrophils. (C) Proportions of immune cell subsets. (D) Expression levels of the marker genes in each subset. (E) Average expression levels of marker transcription factors (TFs) in each subset. (F) Features of marker gene expression in each subset.

To characterize renal immune cells in DIO mice, we performed scRNA-seq on renal cells from DIO KO and WT mice ( Figure 2A). Renal immune cells were enriched prior to sequencing to minimize masking from renal parenchymal cells. The scRNA-seq data yielded a depth of 278,276 reads per cell in WT mice and 197,768 mean reads per cell in KO mice, with 52.60% and 46.30% of reads confidently mapped to the transcriptome, respectively.

After quality control and clustering, we identified 14 subsets of immune cells ( *Cd45* ^+^/ *Ptprc* ^+^) as well as six subsets of intrinsic renal cells, including endothelial cells, mesangial cells, fibroblasts, podocytes, and tubular epithelial cells ( Supplementary Figure S2). Using canonical marker genes [ 5, 7], we annotated immune subsets ( Figure 2B,C), including T cells (T, *Cd3g ^+^*), B cells (B, *Cd79a ^+^*), innate lymphoid cells (ILCs) (NK and ILC1, *Ncr1 ^+^*; ILC2, *Il13 ^+^*), macrophages (M, *Adgre1 ^+^* or *Itgam ^+^*), dendritic cells (cDC1, *Xcr1 ^+^*; pDC, *Siglech ^+^*), and neutrophils (Neu, *Lrg1 ^+^*) ( Figure 2D–F and Supplementary Table S2). T cells comprised the largest proportion of renal immune cells in DIO mice, followed by macrophages, B cells, and NK cells ( Figure 2C).

### *Apoa4* deletion reshapes the renal immune microenvironment

**Figure 3:**
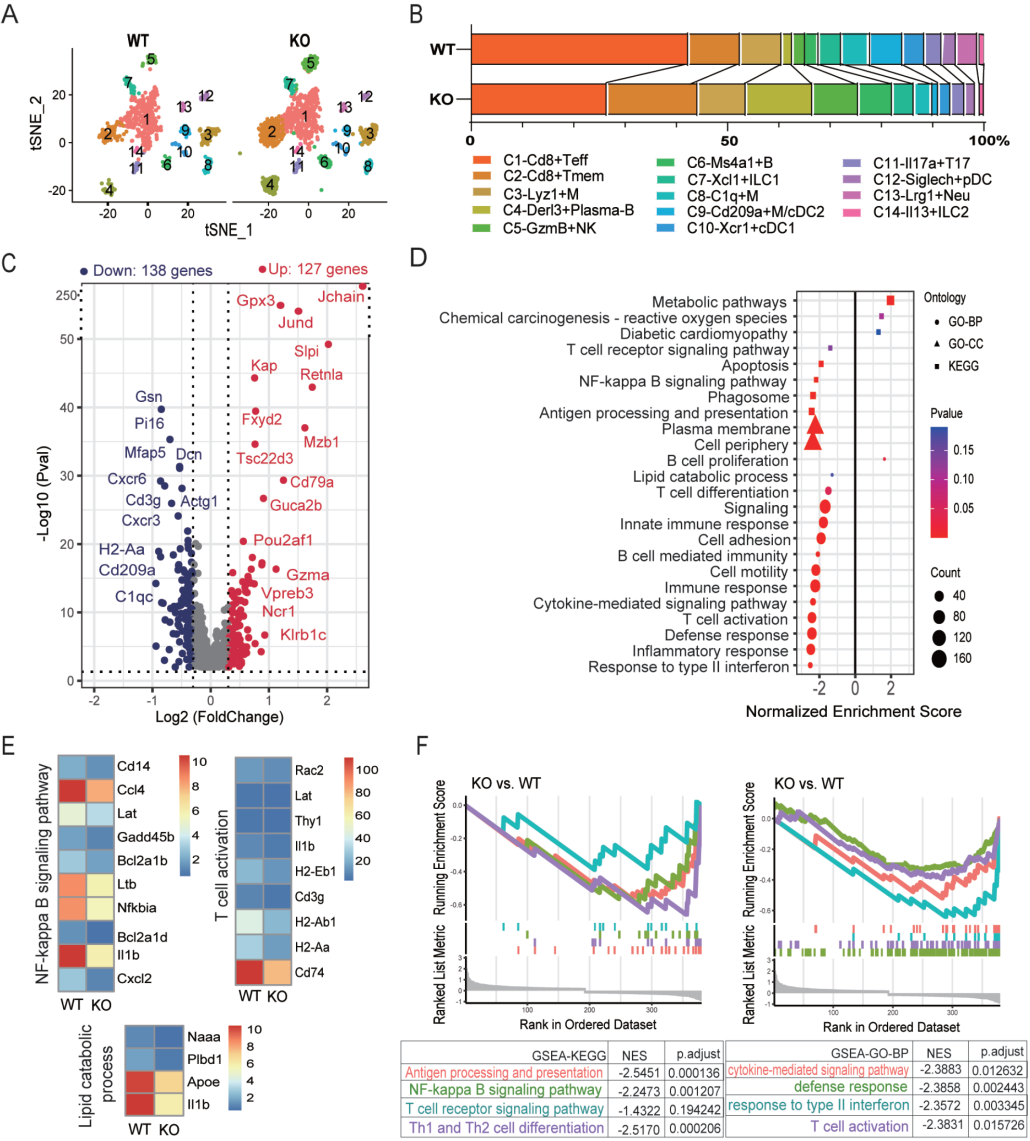
Figure 3 The impact of *Apoa4* deletion on renal immune cells in DIO mice (A) Feature plot of renal immune cells from WT and KO mice. (B) Relative abundance of each subset. (C) Changes in the DEGs of immune cells (KO vs WT). Genes with P adj < 0.05 and |avg_log(fold change)| ≥ 0.3 were defined as DEGs. (D) GSEA of DEGs of immune cells. (E) Average expression levels of key genes involved in biological processes or pathways in (D). (F) GSEA plots of these DEGs. GSEA, gene set enrichment analysis; DEGs, differentially expressed genes; NES, normalized enrichment score; GO, Gene Ontology; BP, biological process; KEGG, Kyoto Encyclopedia of Genes and Genomes.

*Apoa4* deletion changed the renal immune cell composition. Some lymphocyte subsets, such as C5-Gzma ^+^ NK cells, C4-Derl3 ^+^ plasma-B cells, C6-Ms4a1 ^+^ B cells, and C2-Cd8 ^+^ Tmem cells, were increased, whereas the proportions of C1-Cd8 ^+^ Teff cells and some myeloid subsets (C8-C1qc ^+^ M and C9-Cd209a ^+^ M/cDC2) were decreased in KO mice ( Figure 3A,B). Differentially expressed gene (DEG) analysis revealed 127 upregulated and 138 downregulated genes in KO mice compared with WT mice ( *P*_adjust_ < 0.05, |avg_logFoldChange| ≥ 0.3) ( Figure 3C). Notably, the upregulated genes encoded markers of B cells ( *Cd79a*, *Cd79b*), plasma cells ( *Vpreb3*, *Pou2af1*), NK cells ( *Ncr1*, *Klrb1c*, *Gzmb*, *Gzma*), and memory T cells ( *Ccr7*), whereas the downregulated genes included markers of reduced subsets ( *e*. *g*., *Cd3g* for T cells, *C1qc* and *Cd209a* for myeloid cells), suggesting that the cellular composition changes at the transcriptional level ( Supplementary Table S3).

GSEA of the DEGs further revealed downregulated pathways or processes related to immunity, such as T-cell activation, differentiation, antigen processing and presentation, cytokine-mediated signaling pathway, response to type II interferon and immune response in the KO mice and key genes such as *Il1b* , *Ltb*, *Ccl4*, *Cd74* and class II major histocompatibility complex (MHC) molecules, while B-cell proliferation was increased ( Figure 3D–F and Supplementary Table S4). Notably, biological processes, including cell motility and adhesion, and cell components, including the plasma membrane and cell periphery, significantly decreased, suggesting a close relationship between Apoa4 and the cell membrane ( Figure 3D). Furthermore, *ApoA4* deletion was associated with decreased lipid catabolic processes ( Figure 3D), as evidenced by the downregulation of key DEGs, including *Apoe*, *Naaa* , *Plbd1* and *Il1b* ( Figure 3E), alongside upregulated metabolic pathways, chemical carcinogenesis-reactive oxygen species, and diabetic cardiomyopathy ( Figure 3D).

On the basis of the observed alterations in immune cell composition, DEG profiles, and GSEA results associated with *ApoA4* deletion, we focused specifically on its effects on renal lymphocytes in DIO mice.

### *Apoa4* deletion compromises T-cell differentiation and activation

**Figure 4:**
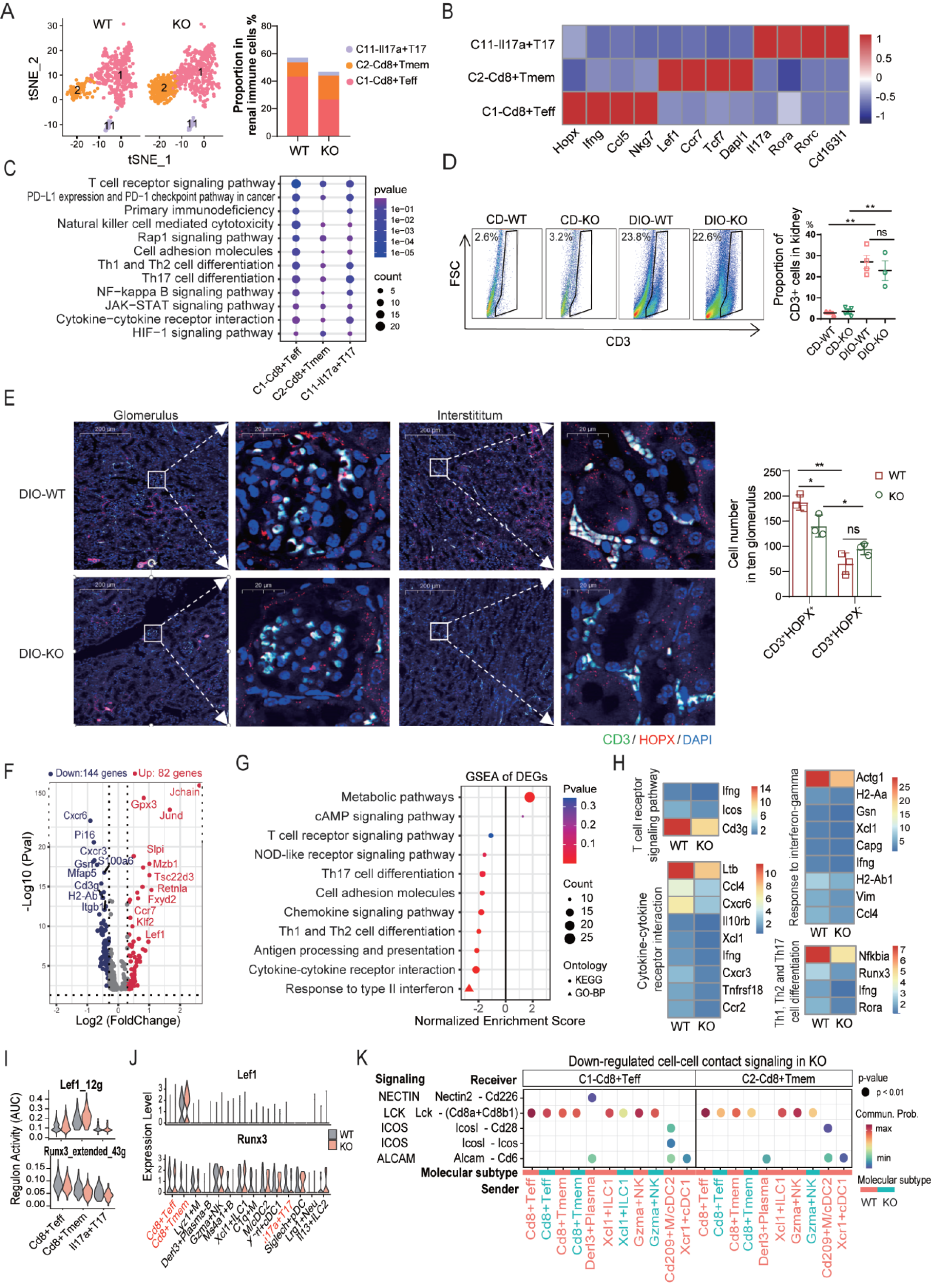
Figure 4 Effects of *Apoa4* deletion on renal T cell (A) tSNE plot and proportions of T-cell subsets. (B) Average expression of marker genes for each T-cell subset. (C) Enrichment analysis of marker genes in each T-cell subset. (D) Representative fluorescence-activated cell sorting (FACS) plots (left panel) and statistical analysis (right panel) of the percentages of CD3+ T cells in the kidney. (E) Immunofluorescence staining (left panel) and statistical analysis (right panel) showing changes in the numbers of CD3+ HOPX+ (representing effector T cells, Teff) and CD3+HOPX – (representing memory T cells, Tmem) cells. (F) Volcano plot of DEGs in total T cells (KO vs WT). A gene is considered significant if P adj < 0.05 and |avg_log(fold change)| ≥ 0.3. (G) GSEA of DEGs from total T cells. (H) Average expression levels of genes involved in key dysregulated pathways. (I) Regulatory network activities of TF Lef1 and Runx3 represented by the area under the curve (AUC) of expression. (J) Expression levels of these TFs. (K) Contributions of key ligand-receptor pairs involved in signaling to C1-Cd8+ Teff and C2-Cd8+ Tmem cells. *P < 0.05; **P < 0.01.

We identified three subsets of T cells ( *Cd3g ^+^Cd3d ^+^*), each characterized by distinct marker genes: C1-Cd8 ^+^ Teff ( *Cd8b1 ^+^Hopx ^+^*), C2-Cd8 ^+^ Tmem ( *Cd8b1 ^+^Lef1 ^+^Hopx ^–^*) and C11-Il17a ^+^ T17 cells ( *Il17a ^+^Rora ^+^*) ( Figure 2C,D, Figure 4A,B and Supplementary Table S2). C1-Cd8 ^+^ Teff cells are characterized by the transcription factor homeodomain-only protein ( *Hopx*), a transcription factor associated with pre-effector T cells progressing toward effector differentiation [35]. Some of these cells expressed cytokines, such as *Ifng* and *Ccl5* ( Figure 4B), indicating potentially activated and late activated T lymphocytes, respectively. Enrichment analysis of marker genes revealed that C1-Cd8 ^+^ Teff cells are involved in pathways related to T-cell receptor signaling, Th1/Th2 and Th17 cell differentiation, natural killer cell-mediated cytotoxicity, the NF-kappa B signaling pathway, and cytokine-cytokine receptor interactions. In contrast, C2-Cd8 ^+^ Tmem cells, marked by *Lef1*, *Ccr7*, and *Tcf7*, were in a relatively quiescent state ( Figure 4C). C11-Il17a ^+^ T17 cells, the smallest subset, expressed *Il17a* and the transcription factor *Rorc* and were enriched in Th17 cell differentiation pathways ( Figure 4B,C).

Frequency analysis revealed that *Apoa4* deletion influenced the overall T-cell population ( Figure 4A), a finding further confirmed by FACS ( Figure 4D). More specifically, the frequency of C1-Cd8 ^+^ Teff cells was reduced, whereas that of C2-Cd8 ^+^ Tmem cells was increased in the KO mice ( Figure 4A). Consistent with these findings, immunofluorescence staining revealed fewer CD3 ^+^HOPX ^+^ cells (representing effector T cells) and more CD3 ^+^HOPX ^–^ cells (representing memory T cells) in DIO KO kidneys than in WT control kidneys ( Figure 4E). The results from the cell cycle analysis revealed that more C1-Cd8 ^+^ Teff cells were arrested in the G1 phase in the KO mice ( Supplementary Figure S3A).

DEG analysis revealed widespread downregulation of genes in T cells from KO mice ( Figure 4F and Supplementary Table S5). GSEA of the DEGs revealed that pathways related to Th1, Th2, and Th17 cell differentiation, as well as immune responses (including cytokine-cytokine receptor interaction, response to type II interferon, and antigen processing and presentation), were significantly downregulated ( Figure 4G and Supplementary Figure S3B). Key genes associated with these pathways, such as *Ifng*, *Il16*, *Ltb*, *Ccl4* , *Cxcr6*, *Cd3g*, *Actg1*, and *MHCII*, were also downregulated ( Figure 4H, Supplementary Figure S3C,D and Supplementary Table S6). SCENIC analysis further revealed alterations in transcription factor (TF) regulatory networks ( Supplementary Figure S3E and Supplementary Table S7). Specifically, in KO mice, the regulatory activity and expression of *Lef1* (a known inhibitor of T-cell development) were upregulated, whereas those of *Runx3* (which promotes cytotoxic T-cell differentiation) [36] were downregulated in the C1-Cd8 ^+^ Teff and C2-Cd8 ^+^ Tmem subsets ( Figure 4I,J and Supplementary Figure S3F). These findings suggest that Apoa4 deficiency is associated with impaired CD8 ^+^ T-cell development and differentiation.

Previous studies have indicated that T-cell activation depends on a metabolic shift from oxidative phosphorylation to glycolysis, whereas quiescent memory cells rely on oxidative phosphorylation [37]. However, in the KO T cells, neither oxidative phosphorylation nor glycolysis significantly changed compared with those in the WT controls ( Figure 4G), suggesting that metabolic reprogramming may not be the primary driver of the observed T-cell phenotype in the Apoa4-deficient mice. To explore alternative mechanisms, we analyzed intercellular signaling using CellChat. This revealed reduced activation and proliferation-related signaling, including ALCAM ( *Alcam-Cd6*) [38], ICOS ( *Icos-Cd28* and *Icos-Icos*), LCK ( *Lck-Cd8* receptor), and NECTIN ( *Nectin2-Cd226*) signaling, in C1-Cd8 ^+^ Teff and C2-Cd8 ^+^ Tmem cells from KO mice ( Figure 4K and Supplementary Table S8). Additionally, GSEA of the DEGs revealed significant downregulation of the plasma membrane, cell periphery, extracellular matrix, and cell adhesion molecules ( Figure 4G), which are critical for signal transduction. These findings collectively suggest that Apoa4 deficiency leads to impaired T-cell differentiation and activation, likely through dampened activation and proliferation signaling and reduced expression of cell periphery components.

### *Apoa4* deletion expands C5-Gzma ^+^ NK cells but impairs effector function and metabolic competence

**Figure 5:**
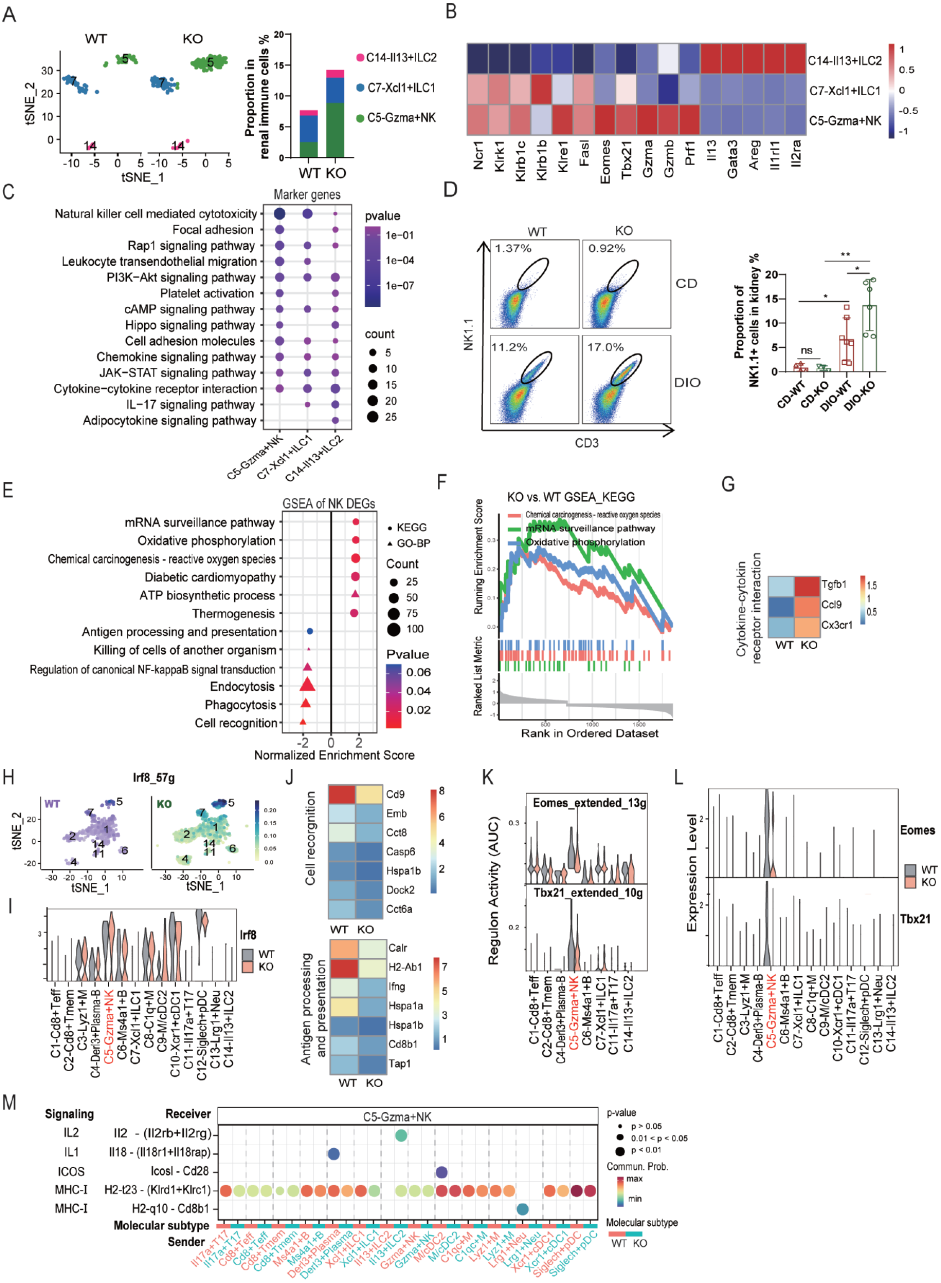
Figure 5 The impact of *Apoa4* deletion on C5-Gzma ^+^ NK cells (A) tSNE plot and proportions of ILC subsets. (B) Average expression of marker genes for each ILC subset. (C) Enrichment analysis of marker genes in each ILC subset. (D) Proportion of NK1.1+ (NK) cells in mouse kidneys was measured by FACS (left panel), and the data in the right panel are statistically significant. * P < 0.05; **P < 0.01. (E) GSEA of DEGs in C5-Gzma+ NK cells. (F) GSEA plot showing upregulated pathways in C5-Gzma+ NK cells. (G) Average expression levels of DEGs involved in cytokine-cytokine receptor interactions in C5-Gzma+ NK cells. (H) Feature plot showing the activity of the regulatory network of the transcription factor Irf8. (I) Expression level of Irf8. (J) Average expression levels of DEGs involved in cell recognition and antigen processing and presentation in C5-Gzma+ NK. (K) Area under the curve (AUC) representing the regulatory network activities of TF Eomes and Tbx21. (L) Expression levels of these TFs. (M) The contributions of ligand-receptor pairs in signaling to C5-Gzma+ NK.

We identified three subsets of innate lymphoid cells (ILCs) in the kidneys: C5-Gzma ^+^ NK ( *Ncr1 ^+^Gzma ^+^*), C7-Xcl1 ^+^ ILC1 ( *Xcl1 ^+^Ncr1 ^+^*) and C14-Il13 ^+^ ILC2 ( *Il13 ^+^Gata3 ^+^*) cells ( Figure 5A,B). C14-Il13 ^+^ ILC2 cells, a minor population expressing the ILC2 markers *Il13* , *Gata3*, *Id2*, *Rora*, and *Areg*, were enriched in cytokine signaling pathways ( Figure 5B,C). The major subsets, C5-Gzma ^+^ NK and C7-Xcl1 ^+^ ILC1 cells, displayed effector functions, expressing both activation receptors ( *e*. *g*., *Ncr1* , *Klrk1*) and inhibitory receptors ( *e*. *g* ., *Klrb1b*, *Klre1*), as well as the apoptotic ligand *Fasl* ( Figure 5B). Notably, C5-Gzma ^+^ NK cells highly expressed the cytotoxic genes *Gzma*, *Gzmb*, and *Prf1* ( Figure 5B) and were significantly enriched in NK cell-mediated cytotoxicity pathways ( Figure 5C), suggesting their potential importance in kidney immunity.

The frequency of C5-Gzma ^+^ NK cells increased in the KO group ( Figure 5A), as validated by FACS analysis ( Figure 5D). GSEA of the DEGs in these cells revealed the upregulation of pathways involved in oxidative phosphorylation, ATP biosynthetic processes and inner mitochondrial membrane protein complexes ( Figure 5E,F and Supplementary Tables S9 and S10), which are pathways linked to increased NK cell proliferation and cytotoxicity [39]. KO NK cells also exhibited elevated expression of *Cx3cr1* ( Figure 5G), a chemokine receptor associated with inflammatory site recruitment. SCENIC analysis further revealed increased regulatory network activity of Irf8 ( Figure 5H,I), a transcription factor known to promote NK cell proliferation [40]. These findings suggest that Apoa4 deficiency promotes the expansion of C5-Gzma ^+^ NK cells, potentially through mechanisms involving metabolic reprogramming, Cx3cr1-dependent recruitment, and Irf8-driven proliferation.

Moreover, GSEA of the DEGs revealed downregulated pathways, including cell recognition, phagocytosis, endocytosis, and regulation of canonical NF-kappaB signal transduction ( Figure 5E), alongside reduced expression of key immune genes, such as *Ifng*, *Cd9*, and *H2-Ab1* ( Figure 5J). The regulatory network activities of *T-bet* and *Eomes*, which are critical for the differentiation and function of NKs [41], were also suppressed ( Figure 5K,L). CellChat analysis further indicated diminished activation signaling, including MHC-I, ICOS, and IL1 signaling, and enhanced immunosuppressive IL2 signaling ( Figure 5M). Collectively, these data suggest that Apoa4 deficiency impairs the effector function of C5-Gzma ^+^ NK cells, likely through dysregulation of the transcription factors *T-bet* and *Eomes*, as well as disruptions in transport processes and signal transduction essential for their activation and differentiation.

Additionally, GSEA revealed upregulation of the mRNA surveillance pathway and chemical carcinogenesis - reactive oxygen species in KO C5-Gzma ^+^ NK cells ( Figure 5E), possibly indicating increased cellular stress responses.

### *Apoa4* deletion increases B-cell abundance but suppresses immune function and metabolic homeostasis

**Figure 6:**
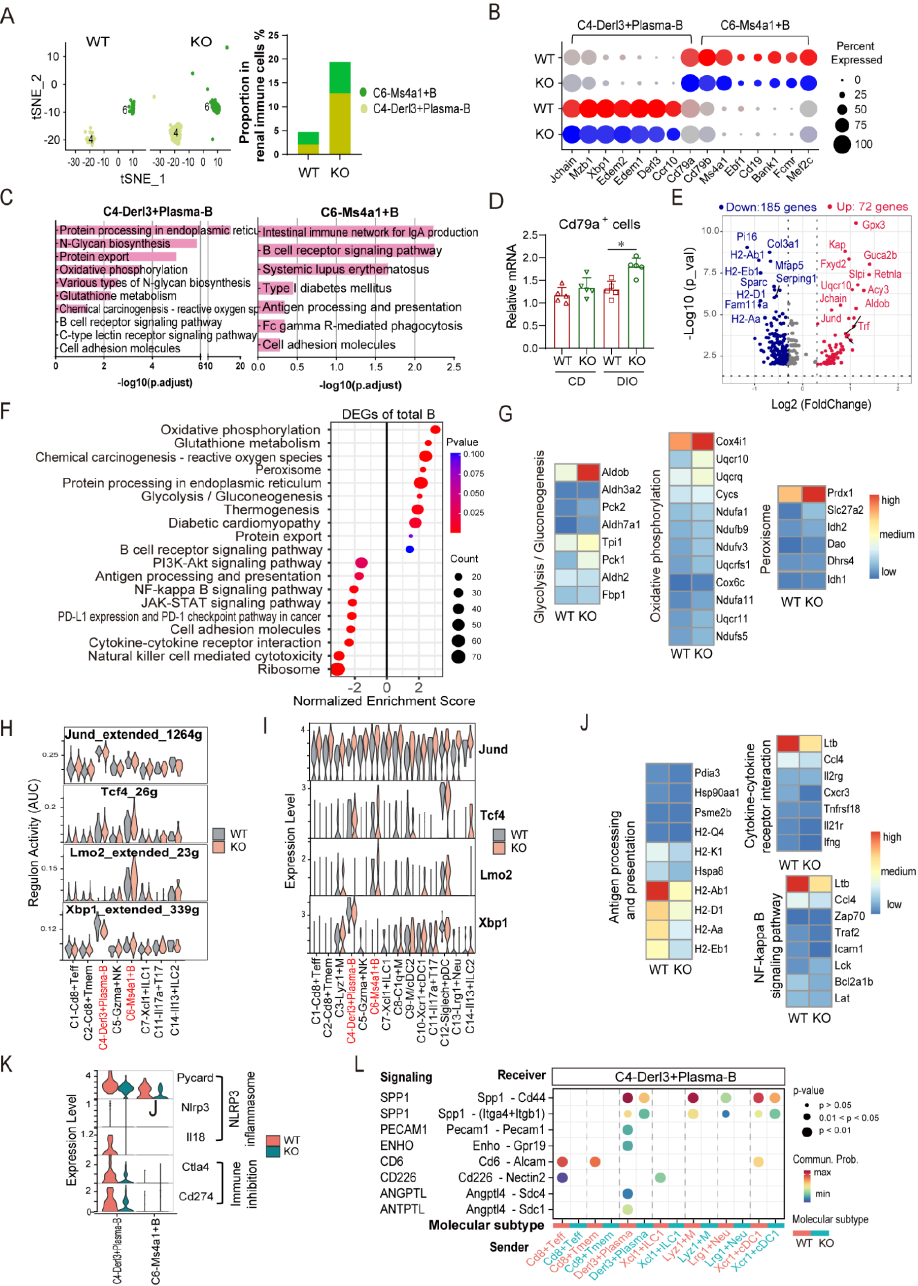
Figure 6 The impact of *Apoa4* deletion on B cells (A) tSNE plot and proportions of B-cell subsets. (B) Expression levels of marker genes in each B-cell subset. (C) Enrichment analysis of marker genes in each subset. (D) Relative expression of Cd79a mRNA in mouse kidneys by qPCR (CD: chow diet). (E) Volcano plot of DEGs in total renal B cells (KO vs WT). Genes with P adj < 0.05 and |avg_log (fold change)| ≥ 0.3 were considered significant. (F) GSEA of DEGs from total B cells. (G) Average expression of DEGs involved in key upregulated pathways in total B cells. (H) Area under the curve (AUC) for regulatory network activities of the TFs Jund, Tcf4, Lmo2 and Xbp1. (I) Expression levels of these TFs. (J) Average expression of DEGs involved in key downregulated pathways in total B cells. (K) Expression levels of NLRP3 inflammasome genes and inhibitory genes. (L) Contributions of ligand-receptor pairs in signaling to C4-Derl3+ plasma-B cells.

We identified two B lymphocyte subsets: C4-Derl3 ^+^ plasma-B ( *Cd79a ^+^Derl3 ^+^*) and C6-Ms4a1 ^+^ B cells ( *Cd79a ^+^Ms4a1 ^+^*) ( Figure 6A,B). C4-Derl3 ^+^ plasma-B cells expressed genes related to antibody production and secretion, including *Jchain*, *Mzb1*, *Xbp1* [42] and *Derl3* ( Figure 6B), and exhibited enrichment in pathways involved in protein processing in the endoplasmic reticulum, N-glycan biosynthesis, protein export and the B-cell receptor signaling pathway, as well as oxidative phosphorylation and glutathione metabolism ( Figure 6C). In contrast, C6-Ms4a1 ^+^ B cells expressed markers associated with immature B cells, such as the development-related transcription factor *Mef2c*, the early B-cell factor-1 *Ebf1* and the pre-B-cell marker *Cd19* ( Figure 6B), and were enriched in the B-cell receptor signaling pathway, the intestinal immune network for IgA production, and autoimmune-related pathways, such as systemic lupus erythematosus ( Figure 6C).

Frequency analysis revealed an increased proportion of B cells in the KO mice ( Figure 6A), which was confirmed by qPCR ( Figure 6D). DEG analysis revealed 72 upregulated genes and 185 downregulated genes in KO B cells ( Figure 6E and Supplementary Table S11). GSEA of the DEGs revealed upregulated enrichment of metabolic pathways ( *e*. *g*., oxidative phosphorylation, glutathione metabolism, glycolysis/gluconeogenesis, peroxisome, and fatty acid metabolic process) ( Figure 6F and Supplementary Table S12) and significant upregulation of peroxiredoxin 1 ( *Prdx1*) and fatty acid transporter ( *Slc27a1*) in peroxisomes ( Figure 6G), which play crucial roles in fatty acid β-oxidation (FAO) and immunometabolism [ 43, 44]. Moreover, B-cell receptor signaling was also upregulated ( Figure 6F and Supplementary Table S12). These upregulated pathways are often associated with B-cell activation and antibody production [45]. SCENIC analysis further revealed elevated regulatory network activity and expression of TF *Jund* [46], *Tcf4* [47], and *Lmo2* [48], which are critical for B-cell development and proliferation, in KO C4-Derl3 ^+^ plasma-B and/or C6-Ms4a1 ^+^ B cells ( Figure 6H,I). Collectively, these findings suggest that Apoa4 deficiency promotes B-cell expansion, possibly through metabolic adaptation and enhanced TF-driven developmental programs.

However, GSEA of the DEGs revealed the downregulation of immune-related pathways, including cytokine-cytokine receptor interaction, antigen processing and presentation, JAK-STAT signaling and lymphocyte activation, alongside reduced expressions of cell adhesion molecules, cell junctions and cell adhesion ( Figure 6F,J, Supplementary Figure S4A and Supplementary Table S12). Similar results were observed in the C4-Derl3 ^+^ Plasma-B and C6-Ms4a1 ^+^ B-cell subsets ( Supplementary Figure S4B,C). For example, KO C4-Derl3 ^+^ plasma-B cells presented reduced antigen processing and presentation, adaptive immune responses, and cell adhesion molecules ( Supplementary Figure S4B). Additionally, the expression levels of NLRP3 inflammasome components ( *Pycard*, *Nlrp3*, and *Il18)* and immune inhibitory genes ( *Ctla4* and *Cd274)* were reduced ( Figure 6K), as were the regulatory network activity and expression of *Xbp1*, a key plasma cell differentiation factor ( Figure 6H,I). CellChat analysis further revealed diminished intercellular signaling to C4-Derl3 ^+^ plasma-B cells, including the activation of SPP1, CD6, and CD226 signaling; the inhibitory signaling ENHO [49]; and the immune homeostasis signaling ANGPTL [50] ( Figure 6L). Collectively, these data indicate that Apoa4 deficiency compromises B-cell immune competence, likely by impairing immune-related pathways and disrupting cell-cell communication. This dysregulation is particularly evident in C4-Derl3 ^+^ plasma-B cells, which exhibit concurrent disruption of both activating and suppressive immune responses.

### The impact of Apoa4 deficiency on macrophages and dendritic cells

Three macrophage subsets were identified: C3-Lyz1 ^+^ M, C8-C1q ^+^ M, and C9-Cd209a ^+^ M/cDC2, each characterized by distinct marker genes ( Figure 2 and Supplementary Figure S5A,B). C3-Lyz1 ^+^ M cells uniquely expressed lysozyme *Lyz1* and the M2-associated marker *Chil3*, whereas C8-C1q ^+^ M cells uniquely expressed the complement genes *C1qb* and *C1qc*, which are linked to pro-resolving phenotypes. C9-Cd209a ^+^ M/cDC2 cells presented dendritic cell-like features, as indicated by the expressions of *Cd209a*, *Flt3*, and *Rbpj*. All subsets expressed pro-inflammatory chemokines such as *Ccl6*, *Ccl9*, and *Il1b* and were enriched in the lysosome and phagosome pathways ( Supplementary Figure S5C), which is consistent with their roles in immune surveillance. In the KO mice, GSEA of the DEGs revealed downregulation of the phagosome, response to type II interferon, antigen processing and presentation, and cell adhesion ( Supplementary Figure S5D), indicating potential immune dysfunction.

Two dendritic cell (DC) subsets were also identified: C10-Xcr1 ^+^ cDC1 cells expressing proliferation markers ( *Mki67*, *Nusap1*, and *Top2a*) with enriched cell cycle pathways and C12-Siglech ^+^ pDC cells expressing *Siglech*, *Atp2a1*, *Cox6a2*, and *Ccr9* ( Supplementary Figure S5A–C). Both subsets exhibited reduced immune-related pathway activity in KO mice ( Supplementary Figure S5D,E), suggesting compromised functionality.

### CellChat analysis shows impaired immune cell-cell interactions in *Apoa4*-deficient mice

**Figure 7:**
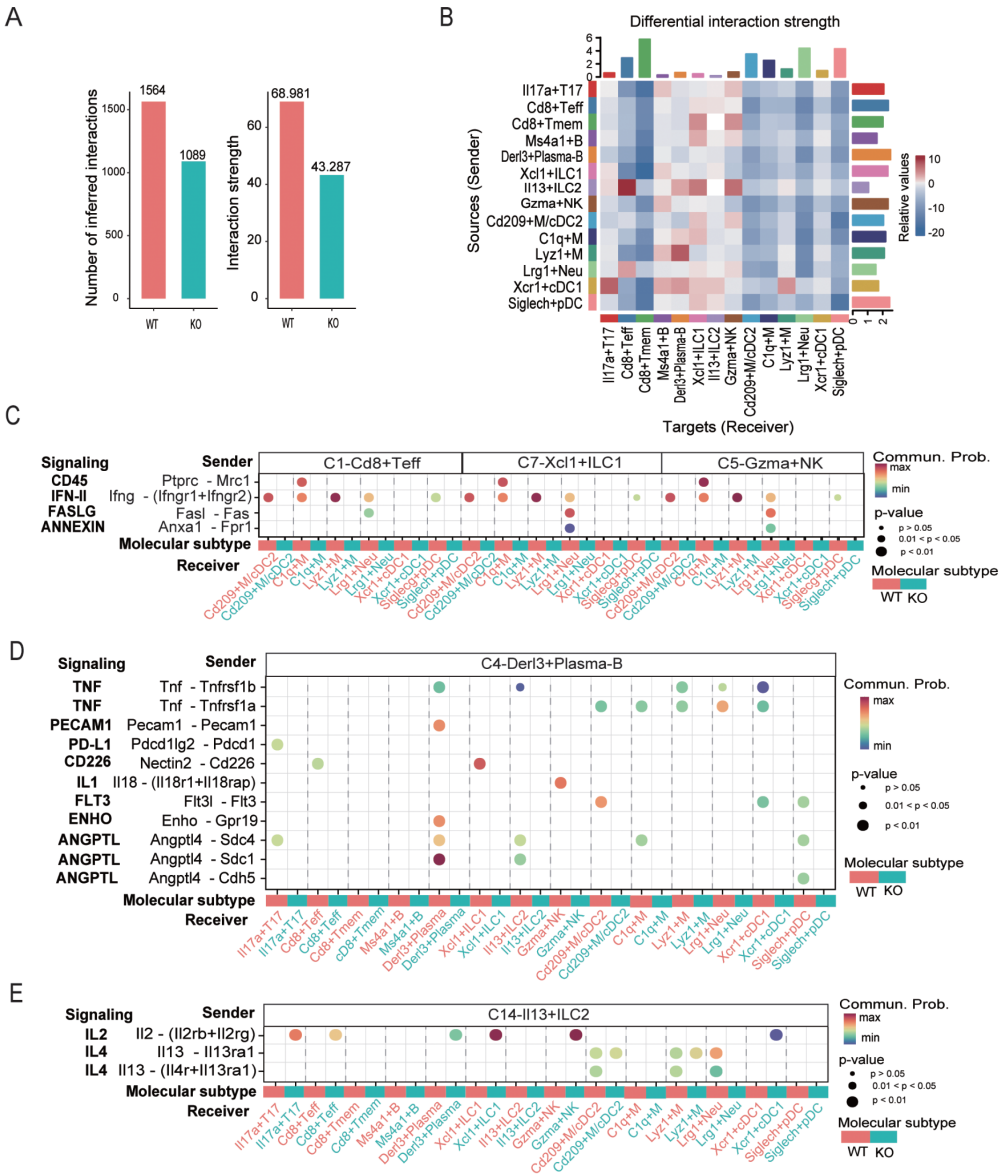
Figure 7 *Apoa4* deletion remodels the renal immune cell-cell interaction inferred by CellChat (A) Number of inferred interactions and interaction strength. (B) Differential interaction strength. (C) Contributions of ligand-receptor pairs in signaling from C1-Cd8+ Teff, C7-Xcl1+ ILC1 and C5-Gzma+ NK to phagocytes. (D) Contributions of ligand-receptor pairs in signaling from C4-Derl3+ plasma-B cells. (E) Contributions of ligand-receptor pairs in signaling from C14-Il13+ ILC2 cells.

Cell-cell interactions are essential for immune regulation. CellChat analysis revealed a global reduction in renal immune communication in KO mice ( Figure 7A), with notably diminished signaling to Cd8 ^+^ Teff, Cd8 ^+^ Tmem, and phagocytes ( Figure 7B), which is consistent with the functional impairments described above. Conversely, signals to Derl3 ^+^ plasma-B and Gzma ^+^ NK cells showed a modest increase; however, these signals originated from cell subsets with low-activity markers ( *e* . *g*., Cd8 ^+^ Tmem, Ms4a1 ^+^ B) or inhibitory Il13 ^+^ ILC2 ( Figure 7B). These data suggest that the expanded Derl3 ^+^ plasma-B and Gzma ^+^ NK cell populations in KO mice receive suboptimal activation signals, potentially contributing to their dysregulated immune responses.

Apoa4 deficiency was associated with reduced lymphocyte-to-phagocyte signaling, including pro-inflammatory signaling IFN-II ( *Ifng*- *Ifngr*), apoptotic signaling FAGLS ( *Fasl*- *Fas*), and suppressive signaling CD45 ( *Ptprc*- *Mrc1*) and ANNEXIN ( *Anxa1*- *Fpr1*) ( Figure 7C). Similarly, Derl3 ^+^ plasma-B cells presented diminished pro-inflammatory signaling by IL1 ( *Il18* - *Il18r*) and TNF ( *Tnf*- *Tnfrsf1b* / *a*), suppressive signaling by PD-L1 ( *Pdcd1lg2*- *Pdcd1*), and cell adhesion signaling by PECAM1 ( *Pecam1*- *Pecam1*), along with altered metabolic signaling by ANGPTL ( *Angptl4*- *Sdc4*/ *Sdc1*/ *Cdh5*) and ENHO ( *Enho-Gpr19*) ( Figure 7D). Il13 ^+^ ILC2s presented reduced IL4 signaling (potentially affecting M2 polarization) and increased IL2 signaling (a known suppressor of lymphocyte activity) ( Figure 7E). In conclusion, Apoa4 deficiency is correlated with broad changes in intercellular signaling, which may dysregulate immune activation and suppression.

## Discussion

Previous scRNA-seq studies characterized diverse renal immune cell populations in both healthy and diseased kidneys, including diabetic kidney disease (DKD) [7], lupus nephritis (LN) [51], fibrosis [52], and renal inflammation [6]. The balance between inflammatory and anti-inflammatory mediators critically influences tissue damage versus repair outcomes. In obesity, this immune equilibrium is often disrupted. Our data, combined with those of prior reports, suggest that Apoa4 deficiency may further aggravate metabolic dysfunction and oxidative stress, potentially dysregulating both pro- and anti-inflammatory responses in early obesity-related CKD.

During acute and chronic kidney injury, T lymphocyte recruitment and activation precede macrophage infiltration. Resident memory T cells and NK cells can rapidly respond to specific antigens, acquiring effector phenotypes to activate innate immunity [ 53 , 54]. Single-cell studies have revealed a lymphocyte-dominated immune landscape in DKD [ 7, 55]. Consistent with these findings, our data from DIO mice revealed that T cells represent the most abundant immune population, followed by macrophages, NK cells, and B cells. Apoa4 deficiency alters lymphocyte composition and functional states, suggesting its potential role in maintaining renal immune homeostasis.

Memory CD8 ^+^ T cells primarily utilize fatty acid β-oxidation (FAO)-driven OXPHOS for survival, whereas effector functions rely on glycolysis. In DIO mice, Apoa4 deficiency is correlated with disrupted T-cell activation signals and metabolic activity, potentially contributing to CD8 ^+^ T-cell dysfunction. B-cell proliferation and antibody production depend on enhanced glycolysis, OXPHOS and B-cell receptor activation [56]. Additionally, B-cell activation depends on co-stimulatory and cytokine signals [45]. B cells in Apoa4-deficient DIO mice show upregulated metabolic (OXPHOS/glycolysis) and B-cell receptor signaling pathways, which coincides with population expansion. However, impaired IFN-γ and TNF signaling suggests functional dysregulation despite increased numbers of B cells. NK cell function is subject to tissue-specific dysregulation in obesity: while peripheral and splenic NK cells exhibit impaired cytotoxicity and IFN-γ production [ 57– 59], adipose NK cells expand and secrete more pro-inflammatory cytokines that exacerbate metabolic dysfunction and inflammation [60]. Hormonal imbalances in obesity, such as short-term leptin activation and long-term suppression of NK cell function, further modulate their activity. In Apoa4-deficient mice, NK cells exhibit elevated expressions of OXPHOS pathway genes and IL-2 signaling markers, potentially facilitating proliferation. Conversely, reduced IL-18 and increased IL-2-associated genes may create an environment less conducive to IFN-γ production, which is consistent with previous findings [ 61, 62]. These data suggest a potential dichotomy in which Apoa4 deficiency may promote cell expansion while limiting their effector function.

Mechanistically, how Apoa4 integrates metabolic and transcriptional regulation, particularly through lipid signaling, warrants further investigation. While the current study is limited by relatively modest cell capture numbers in scRNA-seq, the high sequencing depth (averaging 278,276 reads/cell in WT and 197,768 in KO mice) ensures robust detection of low-abundance transcripts. Moreover, our conclusions remain valid, as they are derived from well-represented cell populations with adequate cell numbers and independently verified through experimental validation.

In conclusion, this study systematically characterized renal immune cell heterogeneity in DIO mice and revealed significant associations between Apoa4 deficiency and lymphocyte metabolic and immune dysregulation. These findings demonstrate that Apoa4 may serve as a key regulator of metabolic-immune crosstalk, maintaining metabolic and immune homeostasis during early obesity-related CKD. Our work deepens the understanding of the interplay between metabolic pathways, cell signaling, proliferation, and immune function.

## Supporting information

25487Supplementary_figures

25487Supplementary_tables

## Supplementary Data

Supplementary data is available at *Acta Biochimica et Biophysica Sinica* online.
