# Supplementary material for: Single-cell transcriptomics reveals apolipoprotein A4-mediated metabolic-immune reprogramming in lymphocytes during early obesity-related chronic kidney disease: Lymphocyte immunity and metabolism in early obese CKD mice
Source: Acta Biochim Biophys Sin (Shanghai). 2025 Sep 25;57(12):1939–52. doi: 10.3724/abbs.2025171 (PMC12747934; doi:10.3724/abbs.2025171)
Supplement: 25487Supplementary_figures [file 25487Supplementary_figures.docx]

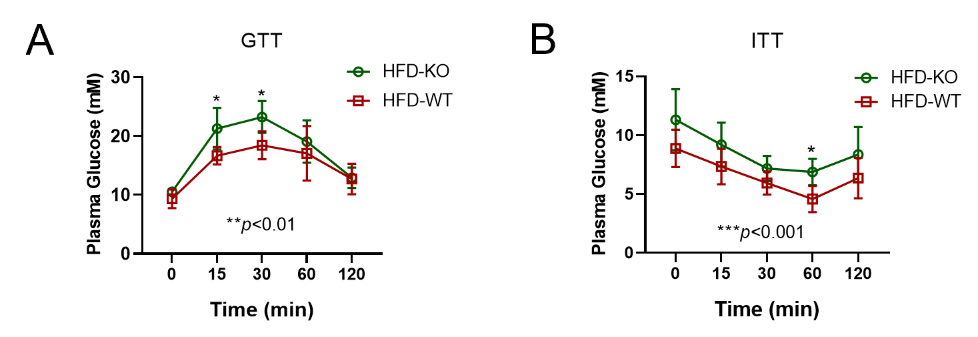


**Supplementary Figure S1. *ApoA4* deletion aggravates insulin resistance (IR) in high-fat diet-induced obese (DIO) mice**  (A) Glucose tolerance test (GTT). (B) Insulin tolerance test (ITT). **P* < 0.05; ***P* < 0.01; ****P* < 0.001; ns: no significant.


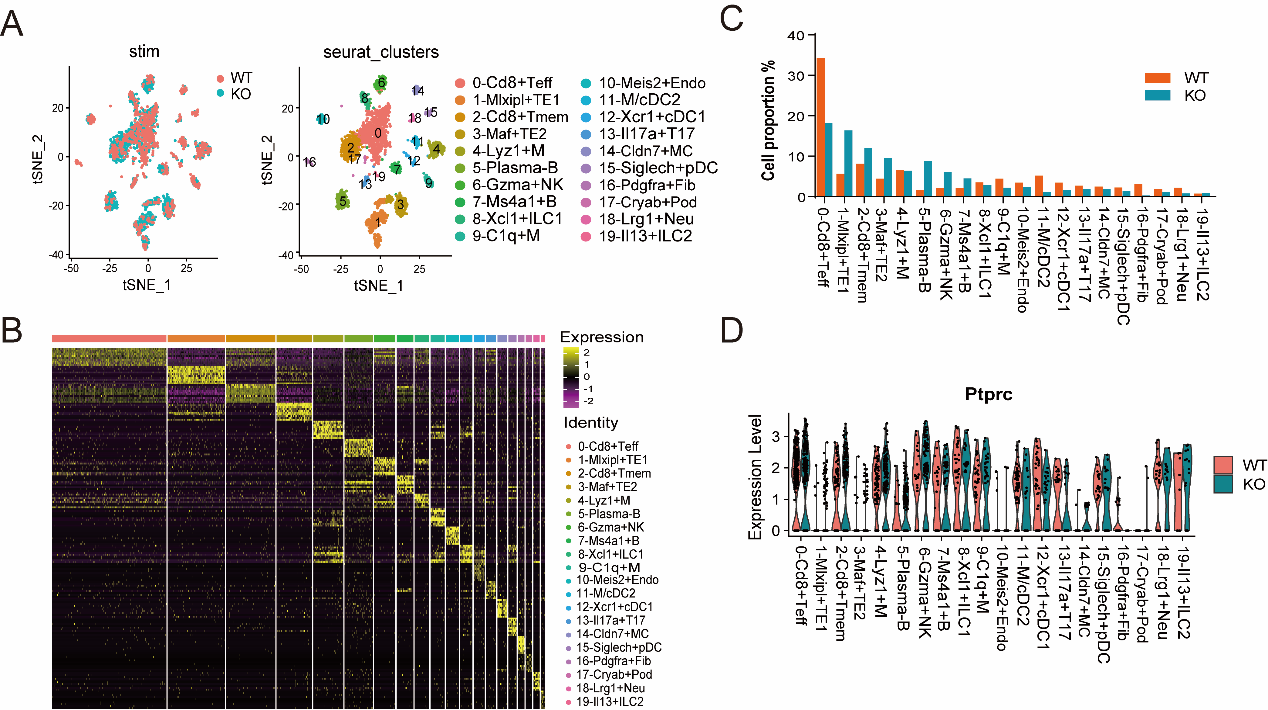


**Supplementary Figure S2. scRNA-Seq identification of kidney cell subsets from DIO mice** (A) t-SNE plot of kidney cells isolated from KO and WT DIO-mice. T: T lymphocytes; Tmem: memory T cells. B: B lymphocytes; ILC: innate lymphoid cells; NK: natural killer cells; M: macrophages; cDC: conventional dendritic cells; pDC: plasmacytoid dendritic cells; Neu: neutrophils. TE: tubular epithelial cells; Endo: endothelial cells; MC: mesangial cells; Fib: fibroblasts; Pod: podocytes. (B) Heatmap showing expression levels of the top 10 marker genes in each subset. (C) Cell proportion in kidney cells. (D) Expression levels of *Ptprc* (*Cd45*) in each subset. KO: *Apoa4*-knockout mice; WT: wild-type mice.


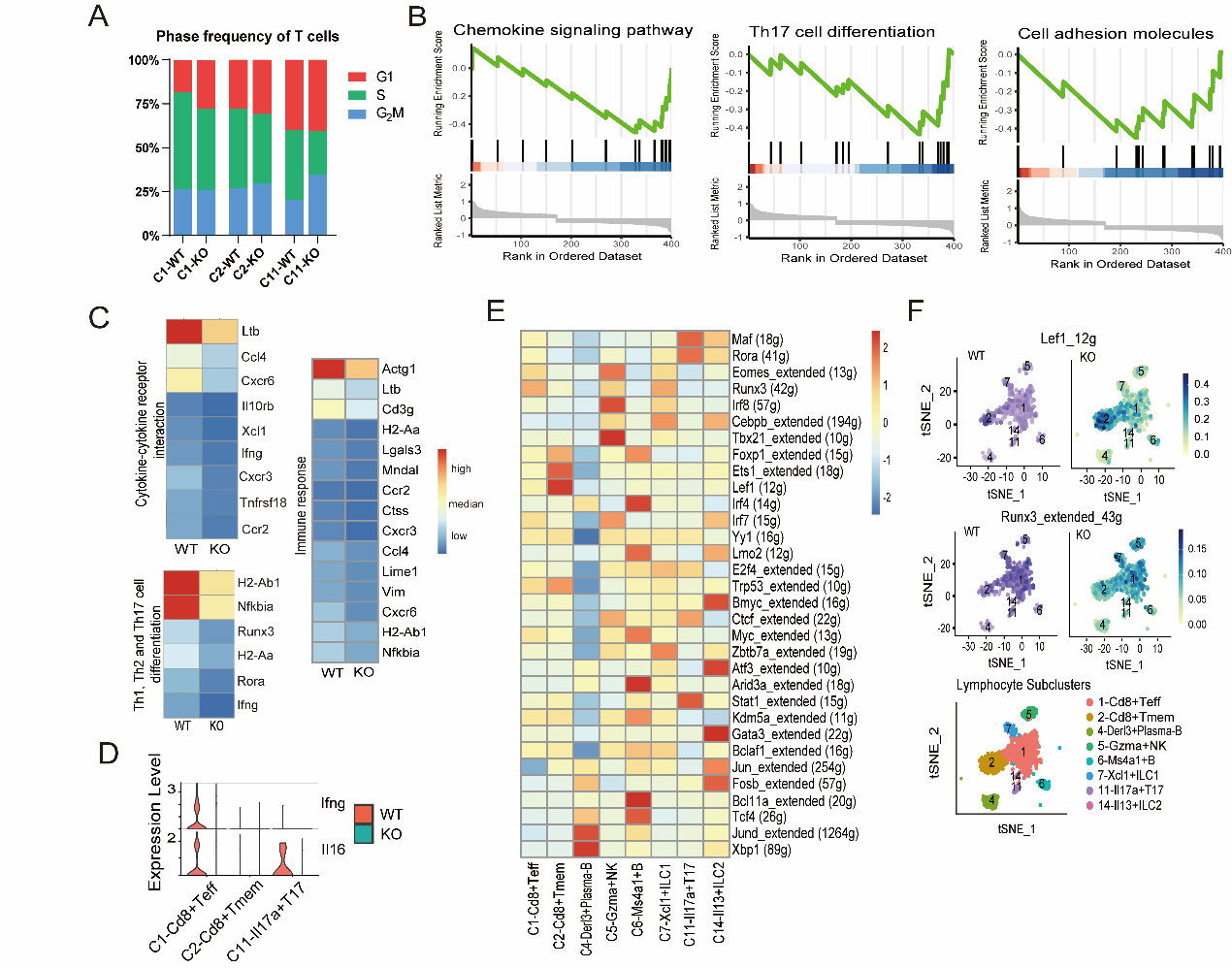


**Supplementary Figure S3. The effects of *Apoa4* deletion on renal T cells** (A) Cell-cycle phase frequency of three T cell subsets. (B) GSEA plot showing KEGG pathways and GO mainly enriched by down-regulated DEGs. (C) Average expression level of genes involved in key KEGG pathways and GO in T cells. (D) Expression levels of Ifng and Il16. (E) Regulatory network activity of TFs in lymphocyte subsets. (F) Feature plot showing Regulatory network activity of *Lef1* and *Runx3* in lymphocytes.

**
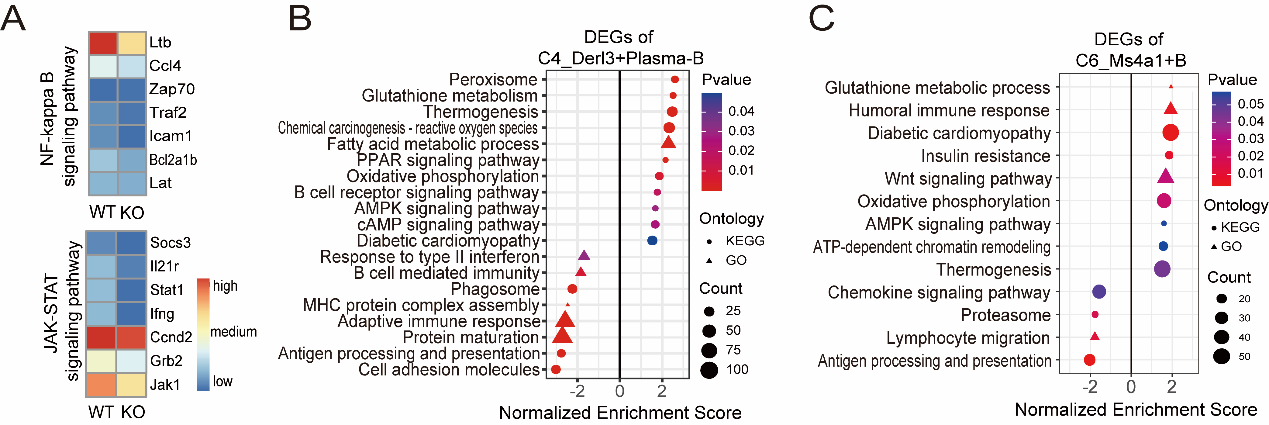
**

**Supplementary Figure S4. The impact of *Apoa4* deletion on renal B cells** (A) The average expression levels of DEGs enriched in immune related pathways in total B cells. (B) GSEA of DEGs of C4-Derl3^+^Plasma-B cells. (C) GSEA of DEGs in C6-Ms4a1^+^ B cells reveals additional pathway alterations linked to Apoa4 deficiency.


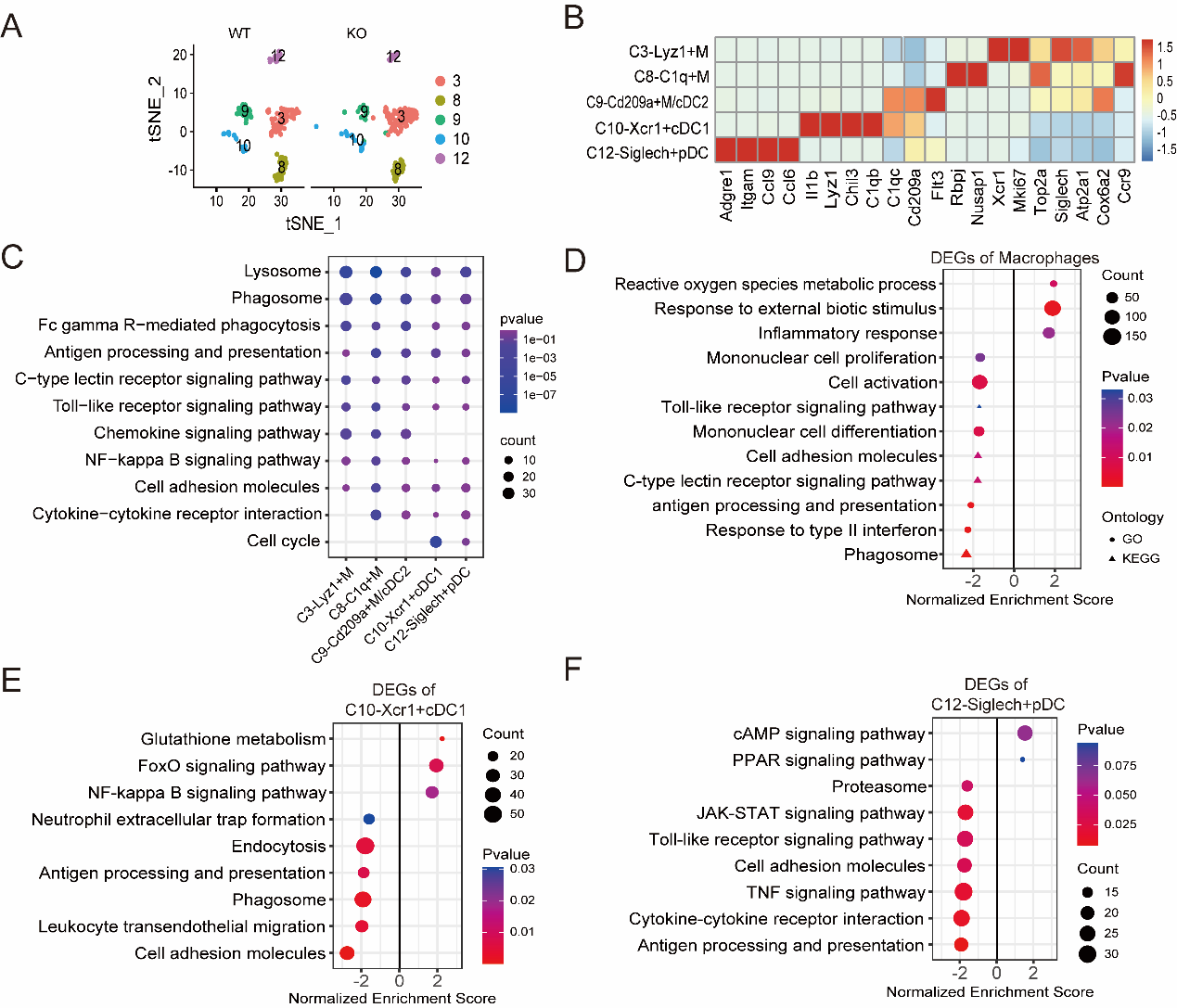


**Supplementary Figure S5. The impact of *Apoa4* deletion on macrophages and DC cells** (A) tSNE plot and proportions of these subsets. (B) Average expression of marker genes. (C) Enrichment analysis of marker genes in each subset. (D) GSEA of DEGs of total macrophages. (E) GSEA of DEGs of C10-Xcr1^+^cDC1. (F) GSEA of DEGs of C12-Siglech^+^pDC.
